# Supplementary material for: Bisulfite treatment and single-molecule real-time sequencing reveal D-loop length, position, and distribution
Source: eLife. 2020 Nov 13;9:e59111. doi: 10.7554/eLife.59111 (PMC7695462; doi:10.7554/eLife.59111)
Supplement: Source code 1. [file elife-59111-code1.rtf.zip › Dloop Length analysis Rscript.rtf]

setwd("/")getwd()library(readr)library(tidyverse)#reading files and shortening the file namefiles <- list.files(".", "bed$")files <- lapply(X = files, FUN = function(x) read_tsv(x, col_names = c("plas", "start", "end", "ZMW", "zero", "strand", "bc_name"))) #normal to see warnings as only some of the many columns are namedfile_names <- list.files(".", "bed$")file_names <- lapply(X = files, FUN = function(x) read_tsv(x, col_names = c("plas", "start", "end", "ZMW", "zero", "strand", "bc_name"))) #normal to see warnings as only some of the many columns are namedfile_names <- gsub("_genePHIX_Pos_50_0.40_CG.PEAK.local.bed.indiv.clust.bed", "", file_names)file_names <- gsub("_bcBC", "", file_names)file_names <- gsub("GC.PEAK.genome.bed", "", file_names)file_names <- gsub(".PEAK.genome.bed", "", file_names)#removing certain parts of the file namefile_names <- gsub("plasmidPHIX_desc", "", file_names)file_names <- gsub("_genePHIX", "", file_names)file_names <- gsub("PCB1811", "", file_names)file_names <- gsub("PCB", "", file_names)file_names <- gsub("_Pos_50_0.4", "", file_names)file_names Start1  <- vector()End1 <- vector()Dlooplength <- vector()for(i in 1:length(files)) {  Start1[I] <- files[[i]] %>% select(start)    End1[I] <- files[[i]] %>% select(end)    Dlooplength [i] <- files[[i]] %>% mutate("D" = end-start) %>% select(D)  df1 <- data.frame(start[i], end[i], UFDlooplength[i]) overlaps[[i]] <- df1}#exporting D-loop length data as excelwrite.table(overlaps[1], “File_name.xls", sep="\t",row.names=FALSE)
