# Supplementary material for: Bisulfite treatment and single-molecule real-time sequencing reveal D-loop length, position, and distribution
Source: eLife. 2020 Nov 13;9:e59111. doi: 10.7554/eLife.59111 (PMC7695462; doi:10.7554/eLife.59111)
Supplement: Source code 2. [file elife-59111-code2.rtf.zip › Position Distribution Analysis Rscript.rtf]

#Analyzing the distribution of D_loop positionsetwd("")getwd()library(GenomicRanges)library(readr)library(tidyverse)plasmid_length <- 2856ranges <- seq(from = 1, to = plasmid_length - 1, by = 1)# make a df that will be turned into a GRanges objects# this df defines the intervals to quantitate overlaps overranges <- seq(from = 1, to = plasmid_length - 100, by = 100)start <- rangesend <- ranges+100-1phix <- data.frame(start=start, end=end)phix$domain_name <- rep("phix", nrow(phix))want <- makeGRangesFromDataFrame(phix,                                  keep.extra.columns=F,                                 ignore.strand=T,                                 seqinfo=NULL,                                 seqnames.field=c("domain_name"),                                 start.field="start",                                 end.field="end",                                 starts.in.df.are.0based=FALSE)#vector_of_overlaps <- countOverlaps(want, bed, type="any")#reading the .bed files obtained from PEAK_GENOME file. For top strand footprint analysis, use ‘*Pos*CG*.bed’ files. files <- list.files(".", "bed$")files <- lapply(X = files, FUN = function(x) read_tsv(x, col_names = c("plas", "start", "end", "ZMW", "zero", "strand", "bc_name"))) #normal to see warnings as only some of the many columns are namedfile_names <- list.files(".", "bed$")file_names <- gsub("_0.4_CH.PEAK.genome.bed", "", file_names)file_names overlaps <- list()for(i in 1:length(files)){  bed <- makeGRangesFromDataFrame(files[[i]],                                  ignore.strand=T,                                  seqinfo=NULL,                                  seqnames.field=c("plas"),                                  start.field="start",                                  end.field="end",                                  starts.in.df.are.0based=FALSE)  vector_of_overlaps <- countOverlaps(want, bed, type="any")  df <- data.frame(start = phix$start,                   end = phix$end,                   depth = vector_of_overlaps,                   perdepth = vector_of_overlaps/sum(vector_of_overlaps),                   fracdepth = vector_of_overlaps/length(vector_of_overlaps))  df$file <- rep(file_names[i], nrow(df))  overlaps[[i]] <- df}#exporting the distribution of position data into a tablewrite.table(overlaps[9],"23_t40w50_CG.xls",sep="\t",row.names=FALSE)#plotting all samples into a big pdfoverlaps2 <- do.call(rbind, overlaps)pdf("all_plots_Line_perdepth_50_sum.pdf", height = 30, width = 30)ggplot(overlaps2, aes(x = start, y = perdepth)) +  geom_line(show.legend=TRUE) +  facet_wrap(~ file)dev.off()#plotting selected files on a graph, showing the D-loop distribution within the region of homologykeep01_05<- c(“A.bed”, “B.bed”) #replace A.bed and B.bed etc with the files you want to plotpdf(“File_name.pdf", width = 5, height = 2)ggplot(overlaps2 %>%          filter(file %in% keep01_05),       aes(x = start, y = perdepth, color=file)) +   ylab("Fraction of D-loops") +  xlab("Reference sequence (bp)") +  geom_point(show.legend=TRUE, cex=2) +  theme(    # Hide panel borders and remove grid lines    panel.border = element_blank(),    panel.background = element_blank(),    axis.line = element_line(colour = "black"),    panel.grid.major = element_blank(),    panel.grid.minor = element_blank()) +#Set xlim based on the region of homology. For each substrate this is different and refer below for the homology windows for each substrate type.  xlim(635, 1566) +  geom_line(cex=1) #+#scale_color_discrete(name = "Substrate") #, labels = c("ds98-915", "ds98-915-78ss"))dev.off()#Use the following  xlim for the respective substratesds98-931:  xlim(635, 1566) ds98-915: xlim(335, 1250)ds98-607: xlim(435, 1042)ds98-197-78ss: xlim(435, 735)
