# Supplementary material for: Bisulfite treatment and single-molecule real-time sequencing reveal D-loop length, position, and distribution
Source: eLife. 2020 Nov 13;9:e59111. doi: 10.7554/eLife.59111 (PMC7695462; doi:10.7554/eLife.59111)
Supplement: Supplementary file 1. — Table summarizing the total number of reads containing a footprint as ‘peak’ and the total number of reads analyzed as ‘total’ for each strand. % Peak’ indicate the percentage of reads containing a footprint. The data represents a cumulation from two independent replicates. [file elife-59111-supp1.docx]

**Supplementary Table 1: Total reads with human proteins**

| **Donor DNA** | **TOP STRAND** | | | **BOTTOM STRAND** | | |
| --- | --- | --- | --- | --- | --- | --- |
|  | **Peak** | **Total** | **% Peak** | **Peak** | **Total** | **% Peak** |
| **Supercoiled donor** | 42 | 2,154 | **2.0** | 19 | 4,898 | **0.39** |
| **Linear donor** | 66 | 2,138 | **3.1** | 8 | 5,104 | **0.16** |
